# Optimistic and ambivalent attitudes toward clinical artificial intelligence

**DOI:** 10.55730/1300-0144.6220

**Published:** 2026-05-16

**Authors:** Sevil UYGUN İLİKHAN, Sinem ÜLKE, Mahmut ÖZER, Matjaž PERC, Yavuz AYHAN

**Affiliations:** 1 Department of Internal Medicine Sciences, Gülhane Faculty of Medicine, University of Health Sciences, Ankara, Turkiye; 2 Department of Internal Medicine, Ankara Bilkent City Hospital, Ankara, Turkiye; 3 Turkish Grand National Assembly, Ankara, Turkiye; 4 Faculty of Natural Sciences and Mathematics, University of Maribor, Maribor, Slovenia; 5 Community Healthcare Center Dr. Adolf Drolc Maribor, Maribor, Slovenia; 6 University College, Korea University, Seoul, Republic of Korea; 7 Department of Physics, Kyung Hee University, Seoul, Republic of Korea; 8 Department of Psychiatry, Hacettepe University, Faculty of Medicine, Ankara, Turkiye

**Keywords:** Artificial intelligence, healthcare professionals, clinical practice, human-artificial intelligence interaction, responsibility, education

## Abstract

**Background/aim:**

Artificial intelligence (AI) is increasingly being integrated into healthcare systems; however, healthcare professionals’ knowledge, attitudes, and perceptions regarding AI remain insufficiently understood. This study aimed to evaluate healthcare professionals’ perceptions of AI in clinical practice.

**Materials and methods:**

This descriptive cross-sectional study included 260 healthcare professionals, including physicians, nurses, and healthcare technicians working at a tertiary public hospital in Türkiye. Participants completed the Healthcare Professionals and Artificial Intelligence Questionnaire, which assessed demographic characteristics, AI knowledge, workplace AI use, attitudes toward AI applications, ethical and legal concerns, perceived professional impact, and educational needs.

**Results:**

Most participants reported limited AI knowledge; 64.6% indicated basic knowledge, whereas 23.1% reported no knowledge. Only 24.6% reported using AI-based systems in clinical practice, while 35.4% were uncertain whether such systems were used in their workplaces. Despite limited experience, attitudes toward recent AI developments were generally positive. Most participants expressed willingness to receive AI-related education (76.9%), although only 6.2% had previously received formal training. Nearly half of the participants believed that the human factor would remain essential in healthcare, whereas a similar proportion considered partial replacement by AI possible. Physicians demonstrated greater confidence in AI usefulness and stronger willingness to integrate AI into clinical decision-making than nurses and other healthcare professionals. Nurses primarily emphasized data security and privacy concerns, whereas physicians highlighted patient safety and misdiagnosis risk. Exploratory psychometric analyses demonstrated a three-factor structure with acceptable sampling adequacy (KMO = 0.765).

**Conclusion:**

Healthcare professionals generally perceive AI as a supportive tool rather than a replacement for human judgment. Limited AI literacy, uncertainty regarding legal responsibility, and insufficient training remain important barriers to responsible AI adoption. Profession-sensitive educational strategies and clearer governance frameworks may support safer AI integration into healthcare practice.

## 1. Introduction

In recent years, artificial intelligence (AI) has transformed the human–machine relationship, moving beyond its former role as a supportive tool toward a more autonomous and influential technological paradigm [1]. In particular, generative AI technologies based on large language models have become capable of performing numerous tasks traditionally carried out by humans by assuming cognitive processes, thereby increasingly reshaping the interaction between humans and intelligent systems [2,3]. While the benefits offered by these technologies are often perceived as significant opportunities, the associated risks are frequently underestimated or overlooked [1,4–6]. Consequently, AI is rapidly influencing and reshaping nearly all domains of life, ranging from education and healthcare to logistics, finance, defense industries, and biotechnology [5,7–11]. The current trajectory of AI development suggests the emergence of increasingly interconnected technological ecosystems, with healthcare constituting one of the most rapidly evolving domains.

In the healthcare sector, the growing volume of digital data flows has created a favorable environment for the rapid integration of algorithm-based clinical decision-support systems into medical practice. AI systems powered by machine learning and deep learning algorithms offer substantial potential to improve diagnostic accuracy and render clinical decision-making processes more data-driven. Particularly in the field of medical imaging, automated image analysis algorithms are widely used to facilitate early disease detection, identify abnormalities, and support radiological reporting [12]. In imaging-based medical specialties, and most notably radiology, the integration of AI into clinical workflows has attracted considerable attention due to its potential to accelerate diagnostic processes, reduce error rates, and alleviate clinicians’ workloads [13]. Beyond medical imaging, AI technologies have expanded into other data-intensive medical fields such as pathology, cardiology, and intensive care medicine, contributing to risk prediction, modeling, patient monitoring, and clinical decision support [14–16]. Collectively, these developments have strengthened expectations that AI-driven systems may play an increasingly influential role in shaping the future of healthcare practice.

Despite this technical potential, the rapid expansion of AI in healthcare has been accompanied by significant ethical, legal, and professional concerns. Issues such as data security, patient privacy, the transparency of algorithmic decision-making processes, and the allocation of responsibility in the event of errors are major challenges to the safe clinical use of AI [17]. Importantly, accumulating evidence suggests that algorithmic risks do not arise solely from biased datasets but may also stem from flawed modeling assumptions embedded within AI systems. For example, Obermeyer et al. demonstrated that a widely used healthcare algorithm exhibited racial bias not due to poor technical performance but because it relied on healthcare expenditures as a proxy for medical need [18]. This algorithmic assumption systematically underestimated the clinical risk of patients with limited access to healthcare, leading to inequitable prioritization despite comparable or greater healthcare needs. Such findings illustrate that even technically high-performing AI systems may produce harmful clinical outcomes when their underlying assumptions fail to reflect real-world healthcare dynamics.

Previous studies have shown that most physicians acknowledge the supportive role of AI systems but remain hesitant to place full trust in automated decision-making processes [19]. Survey-based research further indicates that only a limited proportion of healthcare professionals are highly familiar with AI technologies, while many express concerns regarding potential diagnostic errors, lack of explainability, and unclear accountability structures [20]. These concerns are closely linked to the concept of professional responsibility, as ultimate accountability for medical decisions influenced by algorithmic outputs continues to rest with clinicians. In this context, uncertainties surrounding the boundaries of legal responsibility and the scope of human oversight continue to shape cautious attitudes toward AI adoption.

Recent conceptual studies emphasize that these challenges are closely related to how AI is positioned within healthcare systems. Rather than an automation-driven approach aimed at replacing human judgment, an alternative framework has emerged that emphasizes the complementary use of AI in medicine. Within this framework, AI systems assist healthcare professionals by processing large volumes of data and identifying patterns, while clinical interpretation, ethical judgment, and final decision-making remain under human control [16]. This perspective underscores the necessity of continuous human oversight to ensure that biased assumptions, inappropriate proxies, and context-incongruent recommendations are identified before they result in clinical harm.

Accordingly, differences in levels of AI-related knowledge and awareness among physicians and other healthcare professionals have emerged as a limiting factor in the safe and effective use of these technologies [21]. While the existing literature has largely focused on attitude studies conducted among medical students or physicians [22], comprehensive investigations encompassing nurses and other healthcare workers who play central roles in multidisciplinary clinical workflows remain limited. This gap is particularly important given that AI systems increasingly influence not only diagnostic decisions but also patient management, documentation, and administrative processes.

Despite the potential benefits of AI, healthcare professionals frequently report concerns related to the risk of misdiagnosis, breaches of data privacy, erosion of professional autonomy, and the transformation of clinical roles. To mitigate these risks, numerous studies emphasize the need for targeted and continuous educational programs aimed at enhancing AI literacy, ethical awareness, and critical engagement with algorithmic systems [23]. Beyond technical training, such programs are increasingly conceptualized within participatory and governance-oriented approaches in which healthcare professionals are regarded not merely as end-users of AI but as active stakeholders contributing to the development, evaluation, and oversight of these systems [15].

Against this background, identifying healthcare professionals’ levels of knowledge, attitudes, perceptions, and educational needs regarding AI is of critical importance for the ethical, safe, and clinically meaningful integration of these technologies into healthcare services. Understanding how different professional groups interpret AI-supported decision systems is essential to preventing uncritical automation and to strengthening a human-centered, complementary model grounded in professional judgment. Accordingly, the aim of this study was to systematically examine perceptions of AI among healthcare professionals from different occupational groups and to compare physicians’ attitudes with those of other healthcare professionals. Unlike many previous studies focusing exclusively on physicians or medical students, the present study directly compares physicians and nurses within the same institutional ecosystem, thereby enabling a profession-sensitive evaluation of AI-related perceptions under comparable organizational conditions.

## 2. Materials and methods

### 2.1. Study design and participants

This study was designed as a descriptive cross-sectional survey. It was conducted at Ankara Bilkent City Hospital, a tertiary referral center in Türkiye with a multidisciplinary structure, high patient volume, and active academic training and scientific research activities across multiple medical specialties. The hospital provides advanced healthcare services and employs healthcare professionals from diverse clinical disciplines.

A total of 260 healthcare professionals working at this institution were included in the study. Of the participants, 127 (48.8%) were physicians, 129 (49.6%) were nurses, and 4 (1.5%) were healthcare technicians. The inclusion criteria were defined as being 18 years of age or older, actively working in a healthcare institution, and providing voluntary informed consent to participate in the study. All data were collected by the researchers through face-to-face administration of the questionnaire in the participants’ own clinical units on a voluntary basis. Each participant completed the questionnaire only once, thereby minimizing the risk of duplicate responses.

Ethical approval for the study was obtained from the Ankara Bilkent City Hospital Scientific and Ethical Review Board for Medical Research (TABED; Approval No: TABED 2-25-1381; Date: 6 August 2025). Participation in the study was voluntary, and all data were collected and analyzed anonymously. Although the single-center design may limit the generalizability of the findings, the inclusion of healthcare professionals from multiple clinical disciplines within a large tertiary referral hospital enhances the representativeness of the study sample.

### 2.2. Data collection instrument

Data were collected using the Healthcare Professionals and Artificial Intelligence Questionnaire, which was developed by the researchers based on a focused review of the relevant literature. The questionnaire was constructed through the conceptual adaptation of items commonly used in previous studies examining AI in healthcare. The instrument was designed as a descriptive, multidimensional survey tool. Because the questionnaire was primarily developed as an exploratory descriptive instrument rather than a formal psychometric scale, psychometric analyses were performed to provide preliminary structural and reliability evidence rather than definitive construct validation. The structure of the questionnaire and the distribution of its content domains are summarized in Table 1. To contextualize the instrument and facilitate the interpretation of subsequent analyses, participants’ demographic characteristics and baseline views on AI are presented in Table 2.

The first five items of the questionnaire assessed participants’ descriptive characteristics (sex, age group, type of healthcare institution, professional role, and years of professional experience) and were not included in attitude analyses. Self-reported level of AI knowledge (Item 6), readiness to work with AI systems (Item 11), and views regarding the future of AI in healthcare (Items 14 and 15) were treated as single indicators representing conceptually distinct domains. These variables were defined a priori as high-level indicators and are presented separately in Table 3.

Attitudes and perceptions toward AI were assessed using a broader set of items (Items 9–37), encompassing perceived benefits and risks, ethical concerns, potential effects on professional roles and workload, professional autonomy, and educational needs. These items were analyzed individually and are reported in Table 4.

As an exploratory methodological assessment, internal consistency analyses and exploratory factor analysis were performed for the attitude-related items (Items 9–37). Internal consistency was assessed using Cronbach alpha coefficients, while the underlying structure of the questionnaire was examined using exploratory factor analysis. The suitability of the dataset for factor analysis was evaluated using the Kaiser–Meyer–Olkin (KMO) measure and the Bartlett test of sphericity.

Exploratory factor analysis was performed using principal component extraction with varimax rotation. Factors with eigenvalues greater than 1.0 were retained. Items with factor loadings of ≥0.40 were considered meaningful contributors to the corresponding factor structure.

For clarity, it should be noted that the item numbering presented in Tables 3 and 4 refers only to the AI-related sections of the questionnaire and does not correspond exactly to the complete item numbering of the full instrument. The questionnaire consisted of multiple-choice questions, Likert-type items, and one open-ended question. The Likert-type items were evaluated using predefined response scales. Response options for closed-ended questions are presented in Section 3 where appropriate. An overview of the questionnaire’s overall structure and content distribution is provided in Table 1.

### 2.3. Data collection procedure and statistical analysis

Data were collected by the researchers through face-to-face administration of the questionnaire in participants’ own clinical units based on voluntary participation. Each participant completed the questionnaire only once, and no online or electronic survey methods were used.

Statistical analyses were performed using IBM SPSS Statistics 27.0 (IBM Corp., Armonk, NY, USA). Descriptive statistics were presented as frequencies and percentages for categorical variables and as mean ± standard deviation values for continuous variables. Group comparisons for categorical variables were conducted using the chi-square test. For continuous variables, the independent-samples t-test was applied when parametric assumptions were met, whereas the Mann–Whitney U test was used when these assumptions were not satisfied. Values of p < 0.05 were considered statistically significant.

To account for potential confounding factors, multivariable linear regression analyses were performed. Readiness to work with AI and perceptions regarding the future of AI in healthcare were examined as dependent variables in separate models. Professional role (physicians vs. other healthcare professionals) was included as the primary independent variable, while age, sex, and self-reported level of AI knowledge were entered as covariates. Subgroup analyses by professional category were restricted to physicians and nurses; healthcare technicians were excluded from these analyses due to insufficient sample size. Given the exploratory nature of the item-level analyses, adjustment for multiple comparisons was not performed in order to avoid excessive inflation of type II error. Therefore, the corresponding findings should be interpreted as hypothesis-generating rather than confirmatory.

## 3. Results

A total of 260 healthcare professionals participated in the study. Of the participants, 59.6% were female and 40.0% were male, while 0.4% preferred not to disclose their sex. Regarding age distribution, the majority of participants were in the age group of 26–35 years (75.4%), followed by those aged 18–25 years (17.7%). Most participants were employed by a public hospital (99.2%), with only 0.8% working for university hospitals. In terms of professional roles, 48.8% of participants were physicians, 49.6% were nurses, and 1.5% were healthcare technicians. With respect to professional experience, the largest proportion of participants had 2–5 years of experience (68.1%), followed by those with 6–10 years (16.5%) and ≤1 year of experience (10.8%). Participants’ demographic characteristics and general views regarding AI are presented in Table 2. Due to the insufficient number of healthcare technicians (n = 4) and the predominance of participants employed by a public hospital, comparative analyses were restricted to physicians and nurses, and no comparisons were performed according to type of institution.

Regarding AI knowledge levels, 64.6% of participants reported having a basic level of knowledge about AI, 12.3% reported an advanced level of knowledge, and 23.1% indicated that they had no knowledge of AI. With respect to the use of AI technologies in the workplace, 24.6% of participants reported using AI-based systems, whereas 40.0% reported not using such systems and 35.4% were unsure whether AI-based systems were used in their working environments. When attitudes toward the future of AI were examined, 54.2% of participants evaluated recent AI developments positively and 9.6% very positively. A total of 6.9% of participants believed that AI could completely replace the human factor in healthcare services, whereas 44.6% considered partial replacement possible and 47.3% stated that the human factor would always be necessary.

With regard to AI education, only 6.2% of participants reported having previously received training related to AI; 76.9% expressed a desire to receive such training, while 16.9% reported no interest. The proportion of participants who believed that AI education should be mandatory for healthcare professionals was 38.5%. When readiness to work with AI systems was assessed, 15.4% of participants reported feeling very ready and 30.8% partially ready. In contrast, 36.2% were undecided, 15.0% reported not feeling ready, and 2.7% were definitely not ready.

Item-level analyses of attitudes and perceptions toward AI in healthcare (Items 9–30) are presented in Table 4. When participants were asked about the most important contribution of AI to healthcare services, 38.8% cited reductions in workloads, 27.3% faster diagnoses and earlier interventions, 21.9% reductions in error rates, and 10.0% cost reductions. The most frequently reported areas of AI use were diagnostic and treatment recommendations (16.2%) and medication management and prescribing (13.1%). In contrast, applications in the areas of patient record and management systems (4.2%) and radiology and imaging analyses (8.8%) were reported less frequently. While 32.7% of participants selected “other” areas, 25.0% reported using AI in more than one area.

The most frequently reported concerns related to AI were the risk of misdiagnosis (38.5%) and issues related to patient privacy and data security (33.5%). Physicians reported higher confidence in the usefulness of AI and greater willingness to incorporate AI into clinical decision-making, whereas other healthcare professionals more frequently expressed concerns related to data security and privacy. Differences between professional groups were also observed in perceived benefits, legal responsibility, and educational needs. These findings suggest that physicians tend to evaluate AI primarily through its potential clinical utility, whereas other healthcare professionals place greater emphasis on data governance and patient safety concerns.

Multivariable linear regression analyses were adjusted for age, sex, and self-reported AI knowledge. Being a physician and having higher AI knowledge levels were independently associated with higher readiness to work with AI systems and more positive perceptions regarding the future of AI (Table 3).

All item-level comparisons presented in Table 4 were exploratory in nature and no adjustment was performed for multiple comparisons. Therefore, these findings should be interpreted cautiously and considered hypothesis-generating rather than confirmatory.

**Figure 1:**
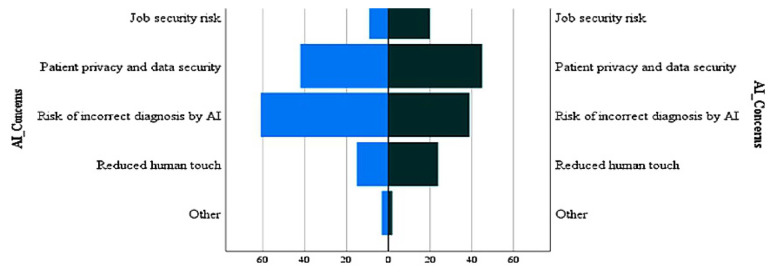
Distribution of perceived concerns regarding artificial intelligence by professional group. This figure illustrates participants’ concerns related to artificial intelligence, categorized as risk of misdiagnosis, patient privacy and data security, reduced human interaction, job security, and other concerns. Group 1 (blue) represents physicians and Group 2 (black) represents other healthcare professionals.

**Figure 2:**
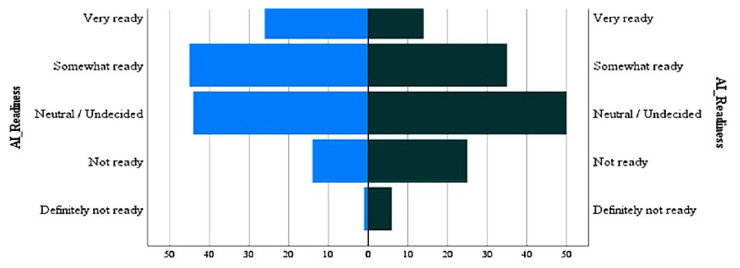
Distribution of readiness to work with artificial intelligence by professional group. This figure depicts participants’ self-reported levels of readiness to work with artificial intelligence, categorized as very ready, partially ready, neutral/undecided, not ready, and definitely not ready. Group 1 (blue) represents physicians and Group 2 (black) represents other healthcare professionals.

**Figure 3:**
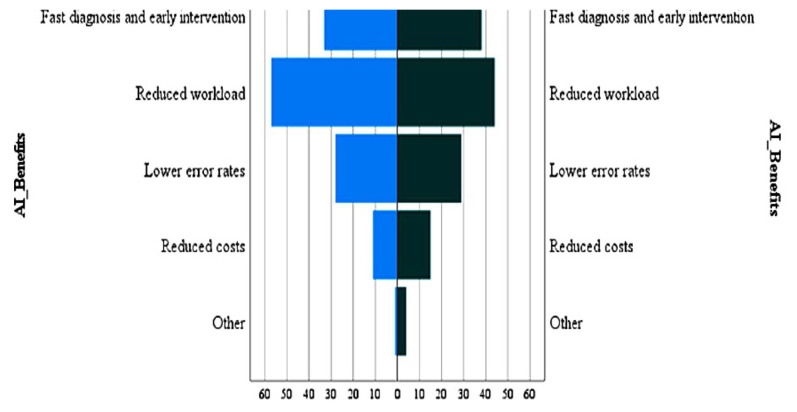
Distribution of perceived benefits of artificial intelligence in healthcare by professional group. This figure illustrates participants’ perceptions regarding the main benefits of artificial intelligence in healthcare, including workload reduction, faster diagnosis, reduced error rates, and cost reduction. Group 1 (blue) represents physicians and Group 2 (black) represents other healthcare professionals.

Exploratory psychometric analyses were performed for the attitude-related questionnaire items. The dataset demonstrated acceptable suitability for factor analysis (KMO = 0.765; Bartlett test of sphericity, p < 0.001). Exploratory factor analysis identified a three-factor structure explaining 45.1% of the total variance (Table 5). Internal consistency analyses demonstrated acceptable reliability for the Perceived Benefit and Role Impact dimensions (Cronbach α = 0.80 for both), whereas the Perceived Risk dimension showed lower internal consistency (Cronbach α = 0.50). Detailed factor loadings are presented in the Supplementary Table. The distribution of perceived concerns regarding AI, readiness to work with AI, and perceived benefits of AI according to professional group are presented in Figures 1–3, respectively. The scree plot supporting factor retention is presented in the Supplementary Figure.

## 4. Discussion

This study provides a profession-sensitive evaluation of healthcare professionals’ knowledge, attitudes, and perceptions regarding the integration of AI into clinical practice.

The psychometric evaluation of the questionnaire identified a three-factor structure with acceptable internal consistency for most dimensions. However, the perceived risk dimension demonstrated relatively low internal consistency. In clinical settings, concerns related to AI encompass diverse domains, including diagnostic accuracy, patient safety, ethical considerations, data privacy, and medicolegal responsibility, which may not strongly correlate within a single dimension. The relatively lower internal consistency observed for the Perceived Risk dimension may reflect the conceptual heterogeneity of ethical, legal, privacy-related, and safety-related concerns associated with clinical AI.

Overall, the findings of this study indicate that AI is generally perceived as a beneficial and supportive tool in clinical practice; however, this positive orientation is accompanied by persistent ethical, legal, and professional concerns. This coexistence of optimism and caution is consistent with previous studies examining AI adoption in healthcare and reflects the complex nature of integrating algorithmic systems into clinical decision-making processes [24].

Beyond this general pattern, an important but less explicitly articulated finding of this study is the high level of uncertainty and ambivalence observed among healthcare professionals. A substantial proportion of participants reported being unsure whether AI systems were used in their workplace and described themselves as undecided regarding their readiness to work with AI. In the literature, such ambivalence is not interpreted as resistance, but rather as an indicator of limited explainability, insufficient institutional communication, and weak integration of AI systems into everyday clinical workflows [19,21]. These findings suggest that the diffusion of AI in healthcare has outpaced the development of shared understanding and professional confidence.

A central finding of this study is the presence of profession-specific differences between physicians and other healthcare professionals. Physicians demonstrated significantly stronger confidence in the usefulness of AI applications and expressed greater willingness to incorporate AI-supported tools into future clinical decision-making. In contrast, nurses and other healthcare professionals reported heightened concerns related to data security, patient privacy, and ethical risks. These differences may be explained by distinct professional roles and epistemic responsibilities within healthcare systems. Physicians are typically positioned as final decision-makers and may therefore perceive AI primarily as a clinical decision-support instrument, whereas other healthcare professionals are more closely involved in data handling, patient interaction, and institutional processes that foreground concerns related to privacy and governance.

These findings align with previous studies suggesting that attitudes toward AI differ across healthcare professions and are shaped by professional responsibility, accountability, and proximity to clinical decision-making processes [25,26]. Importantly, these differences do not necessarily reflect resistance to AI technologies; rather, they indicate variations in how occupational groups evaluate potential risks and benefits. These differences may also reflect varying degrees of direct exposure to diagnostic decision-making processes and differing professional responsibilities regarding patient data management.

Ethical and legal concerns emerged as a dominant theme across all participant groups, particularly regarding responsibility allocation in cases of AI-related error. Physicians were more likely to attribute responsibility to developers or software manufacturers, whereas other healthcare professionals tended to locate responsibility at the level of clinicians and practitioners. This divergence mirrors ongoing international debates on accountability in medical AI and highlights unresolved tensions between technological development and clinical governance [27]. These findings resonate with regulatory and policy discussions emphasizing that accountability gaps undermine trust in AI-enabled clinical systems. From this perspective, ambiguity in legal responsibility not only affects clinicians’ willingness to rely on AI but also reinforces cautious and defensive professional attitudes [28]. Clarifying responsibility frameworks may therefore be as important as improving algorithmic accuracy for sustainable AI integration.

In addition, the relatively high proportion of participants expressing neutrality or indecision toward AI can be interpreted as a transitional state between traditional clinical practice and human–AI collaboration. Similar transitional patterns have been observed in studies examining early-stage AI adoption, where professionals neither reject technology nor fully trust it, but instead await clearer institutional norms and safeguards [29,30]. In other words, although the accelerating integration of AI into healthcare systems is often framed around efficiency gains, the automation of routine tasks, and improvements in diagnostic accuracy, our findings are consistent with recent literature in suggesting that the primary challenge is no longer whether AI can be deployed in medicine but rather how it should be deployed without amplifying existing ethical, legal, and professional risks [4,9].

Another key finding of this study concerns the substantial gap between healthcare professionals’ interest in AI-related education and their actual training experiences. While the majority of participants expressed a willingness to receive AI training, only a small proportion reported having previously received formal instruction. This discrepancy highlights a structural deficiency in current healthcare education systems rather than a lack of individual motivation. Similar gaps have been reported in the literature and are increasingly recognized as obstacles to responsible AI adoption [23].

Notably, the finding that a majority of participants wished to receive AI education while a much smaller proportion supported mandatory training reveals an important tension between individual motivation and institutional regulation. This pattern suggests that healthcare professionals recognize AI as necessary for future practice but remain cautious about top-down enforcement, possibly due to concerns over additional workload, inadequate training quality, or unresolved responsibility issues [23,29]. Importantly, the need for AI education extends beyond technical proficiency. Participants’ concerns about explainability, liability, and erosion of professional autonomy suggest that educational initiatives must also address ethical reasoning, legal frameworks, algorithmic bias, and human–AI interaction. In this context, AI literacy should be understood as a multidimensional competency that enables healthcare professionals to engage critically with AI systems rather than defer unreflectively to algorithmic outputs. This perspective aligns with participatory and society-in-the-loop governance approaches, which emphasize shared responsibility and continuous evaluation of AI systems throughout their lifecycles [15,16]. From a practical perspective, successful AI integration in healthcare may require profession-sensitive educational programs, transparent governance frameworks, and institutional policies that clarify responsibility allocation and support explainable AI use in clinical workflows.

Taken together, while the present findings provide important insights into healthcare professionals’ perceptions of AI, several limitations should be considered while interpreting the results. The cross-sectional nature of the study limits causal inferences and does not allow for examination of how attitudes and perceptions may change as AI technologies become more deeply integrated into clinical workflows. In addition, the study was conducted in a single tertiary healthcare institution, which may reduce the generalizability of the findings to settings with different organizational structures, technological capacities, or governance frameworks. Furthermore, the study population predominantly consisted of young healthcare professionals employed by a public healthcare institution, which may limit the transferability of the findings to private healthcare systems, senior professionals, or institutions with different organizational cultures or technological infrastructures. In addition, potentially relevant variables such as departmental workload, prior digital technology exposure, institutional AI infrastructure, and specialty-specific clinical experiences were not included in the regression models and may have influenced participants’ perceptions. Therefore, future research would benefit from multicenter and longitudinal designs that track changes in perceptions over time and across diverse healthcare contexts. In addition, although psychometric analyses were performed, further validation studies in different populations are warranted. Furthermore, the exploratory psychometric findings should be interpreted cautiously, as the questionnaire was originally designed as a descriptive research instrument rather than a standardized psychometric scale.

## Supplementary materials

Supplementary Figure Scree plot of the exploratory factor analysis.
The scree plot supports the retention of a three-factor structure.

**Supplementary Table:** Rotated factor loadings of the exploratory factor analysis.

Item	Perceived benefit	Role impact	Risk
**Item** 9	0.673		
**Item** 10	0.678		
**Item** 11	0.681		
**Item** 12	0.756		
**Item** 13	0.756		
**Item** 2	0.495		
**Item** 6		0.576	
**Item** 7		0.626	
**Item** 14		0.462	
**Item** 15		0.624	
**Item** 17		0.601	
**Item** 18		0.663	
**Item** 19		0.768	
**Item** 20		0.527	
**Item** 21		0.681	
**Item** 3			0.486
**Item** 4			0.556
**Item** 5			0.604
**Item** 16			0.571

Only factor loadings ≥0.40 are displayed for clarity.

Item numbering corresponds to the coding structure used during the exploratory factor analysis and may not exactly match the sequential numbering of the complete questionnaire.

## Figures and Tables

**Table 1:** Healthcare Professionals and Artificial Intelligence Questionnaire: item list and content distribution.

A) Descriptive characteristics (not part of the AI-related questionnaire content).	
Item No.	Question
1	Sex
2	Age group
3	Type of healthcare institution
4	Professional role
5	Years of professional experience
**B)** Artificial intelligence-related questionnaire items.	
6	Do you have knowledge about artificial intelligence (AI) technologies?
7	Are AI-supported systems used in your working environment?
8	If yes, in which areas do you use AI? (Multiple options may be selected)
9	What do you consider to be the greatest benefit of AI in healthcare?
10	What is your greatest concern regarding the widespread use of AI in healthcare?
11	To what extent do you feel prepared to work with AI systems?
12	Have you received any training related to the use of AI?
13	Do you think AI education should be mandatory for healthcare professionals?
14	How do you evaluate recent developments in AI in the healthcare sector?
15	Do you think AI may replace the human factor in healthcare services in the future?
16	Would you like to share additional views regarding the future of AI in healthcare?
17	Do you think the role of AI in medicine is sufficiently understood and implemented?
18	Do you think AI projects increase efficiency in healthcare services?
19	Do you think the use of AI in medicine will lead to ethical problems?
20	Do you think AI applications in healthcare should be comprehensively evaluated, ethically reviewed, and formally authorized?
21	Do you think physicians need more training to use AI-based tools in clinical practice?
22	Do you think AI will fundamentally change physicians’ roles in medicine?
23	Do you think AI will replace many physicians?
24	Do you think AI can be ignored in medicine?
25	Do you think AI applications will support healthcare professionals?
26	Do you think AI will enhance the professional experience of healthcare professionals?
27	Do you think AI will reduce workload and burnout among healthcare professionals?
28	Do you think AI products will enable more efficient healthcare delivery at lower cost?
29	Do you think AI will reduce workload, increase time spent with patients, and improve quality of care?
30	Do you think the introduction of AI into medicine will create new ethical problems and make physicians’ work more complex?
31	Do you think AI products will reduce physicians’ income?
32	Do you think AI-focused medical education aimed at improving physicians’ AI competencies should be accessible to all physicians?
33	Do you think long-term use of AI will weaken physicians’ clinical experience and decision-making skills, thereby negatively affecting medical education?
34	Do you think the use of AI will reduce physicians’ professional autonomy?
35	Do you think the use of AI will reduce physicians’ professional status?
36	Do you think AI products will increase physicians’ workload?
37	Do you think AI will displace physicians from their professional positions?

**Table 2:** Demographic characteristics and artificial intelligence-related views of the participants (N = 260).

Variable	Category		%
Sex	Female	155	59.6
	Male	104	40.0
	Prefer not to say	1	0.4
Age group (years)	18–25	46	17.7
	26–35	196	75.4
	36–45	15	5.8
	46–55	3	1.2
Type of institution	Public hospital	258	99.2
	University hospital	2	0.8
Professional role	Physician	127	48.8
	Nurse	129	49.6
	Healthcare technician	4	1.5
Years of professional experience	≤1 year	28	10.8
	2–5 years	177	68.1
	6–10 years	43	16.5
	≥11 years	12	4.6
Level of AI knowledge	Advanced	32	12.3
	Basic	168	64.6
	None	60	23.1
Use of AI at the workplace	Yes	64	24.6
	No	104	40.0
	Not sure	92	35.4
Attitude toward recent AI developments	Very positive	25	9.6
	Positive	141	54.2
	Neutral	89	34.2
	Negative	5	1.9

Healthcare technicians (n = 4) were included for descriptive purposes only and were excluded from comparative analyses due to insufficient representation.

**Table 3:** Multivariable linear regression analyses for key attitudinal indicators toward artificial intelligence.

Predictor	Readiness to work with AI B (95% CI)	p-value	Perceptions of the future of AI B (95% CI)	p-value
**Physician (vs. other healthcare professionals)**	0.323 (0.09–0.55)	0.006	0.230 (0.08–0.38)	0.003
**Sex**	−0.094 (−0.32 to 0.13)	0.420	0.010 (−0.14 to 0.16)	0.896
**Age group**	0.083 (−0.13 to 0.30)	0.452	0.076 (−0.07 to 0.22)	0.300
**Level of AI knowledge**	0.629 (0.44–0.82)	<0.001	0.378 (0.25–0.51)	<0.001

AI: Artificial intelligence; CI: confidence interval.

Note: B coefficients indicate the adjusted change in the dependent variable. Positive values indicate higher scores, whereas negative values indicate lower scores. Reference categories are shown in parentheses.

**Table 4:** Exploratory item-level comparison of artificial intelligence-related attitude items between physicians and other healthcare professionals.

Question item	Mean difference	95% CI (lower)	95% CI (upper)	p-value
Item 9	0.077	−0.113	0.267	0.426
Item 10	−0.231	−0.420	−0.042	0.017
Item 11	−0.100	−0.330	0.130	0.393
Item 12	−0.200	−0.407	0.007	0.058
Item 13	−0.208	−0.395	−0.020	0.030
Item 14	−0.069	−0.303	0.165	0.560
Item 15	0.200	−0.036	0.436	0.097
Item 17	0.531	0.329	0.732	<0.001
Item 18	−0.162	−0.297	−0.026	0.020
Item 19	−0.262	−0.461	−0.062	0.010
Item 20	−0.385	−0.611	−0.158	0.001
Item 21	−0.238	−0.455	−0.022	0.031
Item 22	−0.146	−0.357	0.064	0.173
Item 23	0.008	−0.230	0.246	0.949
Item 24	−0.131	−0.367	0.106	0.278
Item 25	−0.308	−0.498	−0.117	0.002
Item 26	0.015	−0.231	0.261	0.902
Item 27	−0.008	−0.247	0.231	0.949
Item 28	0.038	−0.195	0.272	0.746
Item 29	0.162	−0.053	0.376	0.140
Item 30	−0.054	−0.298	0.190	0.664

The analyses were exploratory in nature and no adjustment for multiple comparisons was performed. Therefore, p-values should be interpreted cautiously and primarily as hypothesis-generating findings rather than confirmatory evidence.

**Table 5:** Exploratory factor analysis and internal consistency results of the questionnaire dimensions.

Factor	Variance explained (%)	Cronbach’s α
**Perceived benefit**	22.2	0.80
**Role impact**	14.6	0.80
**Perceived risk**	8.3	0.50

Kaiser–Meyer–Olkin measure = 0.765; Bartlett test of sphericity, p < 0.001.
