# Supplementary figures and images for: Optimistic and ambivalent attitudes toward clinical artificial intelligence
Source: Turk J Med Sci. 2026 May 16;56(3):860–71. doi: 10.55730/1300-0144.6220 (PMC13398542; doi:10.55730/1300-0144.6220)

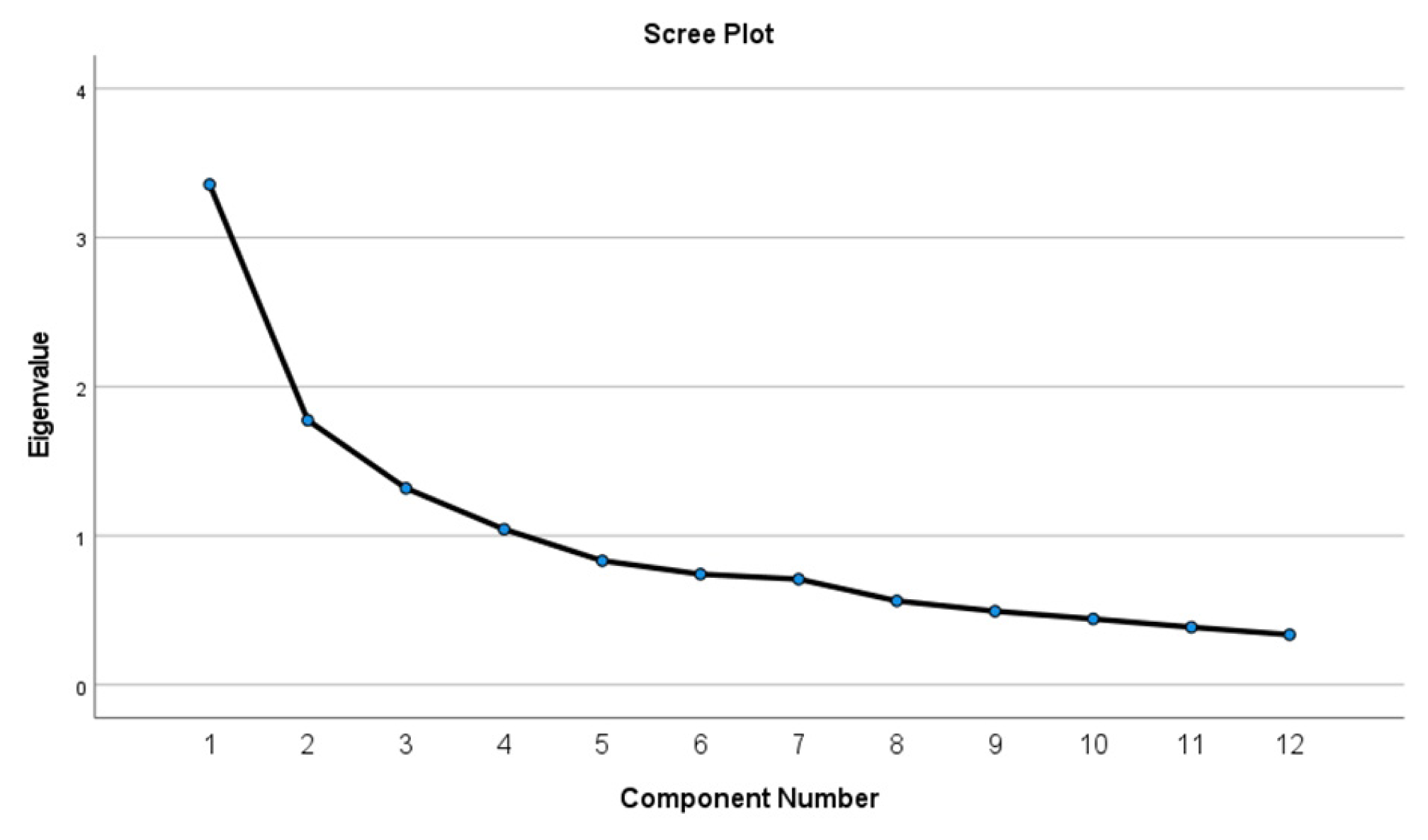

Supplement: Supplementary Figure — Scree plot of the exploratory factor analysis. The scree plot supports the retention of a three-factor structure. [file tjmed-56-03-860s1.tif]
